# Nutrition in Advanced Thyroid Cancer Patients

**DOI:** 10.3390/nu14061298

**Published:** 2022-03-18

**Authors:** Laura Agate, Elisa Minaldi, Alessio Basolo, Valentina Angeli, Roberta Jaccheri, Ferruccio Santini, Rossella Elisei

**Affiliations:** 1 Endocrinology Unit, Department of Clinical and Experimental Medicine, University Hospital of Pisa, 56124 Pisa, Italy; laura.agate@virgilio.it (L.A.); elisa.minaldi@phd.unipi.it (E.M.); alessio.basolo@med.unipi.it (A.B.); r.jaccheri@ao-pisa.toscana.it (R.J.); ferruccio.santini@med.unipi.it (F.S.); 2 Dietary Service, University Hospital of Pisa, 56124 Pisa, Italy; valentina.angeli@ao-pisa.toscana.it

**Keywords:** thyroid cancer, multikinase inhibitors, nutritional therapy

## Abstract

In the last decade, multikinase inhibitors (MKIs) have changed the paradigm of treatment of advanced and progressive thyroid cancer. Compared with the traditional treatment with chemotherapy and radiotherapy, these new drugs have shown a good efficacy in controlling the neoplastic disease, and also a different toxicity profile compared to traditional chemotherapy, milder but still present and involving mainly the nutritional profile. Weight loss, nausea, anorexia, stomatitis, diarrhea may be associated with malnutrition and cancer-related cachexia. The latter is characteristic of the advanced cancer stage and may be present before starting MKIs, or may develop afterwards. Adverse events with nutritional impact may cause a significant impairment of quality of life, often requiring dose reduction and sometimes drug discontinuation, but with a lower efficacy on the neoplastic disease. The aim of this paper was to discuss the role of nutritional therapy in advanced thyroid cancer and the importance of prevention, early recognition and careful management of malnutrition and cachexia during systemic therapy with MKIs.

## 1. Introduction

Well-differentiated thyroid carcinoma (TC) is one of the human tumors with the best prognosis, achieving a 5-year survival rate of 98.3% and only rarely reaching an advanced stage of the disease [1]. However, local recurrences occur in about 20% of cases and distant metastases in 10%, more commonly in the lung (50%) and bone (25%) [2]. The outcome of these advanced tumors remains favorable as long as they respond to radioiodine (RAI) treatment [3]. In 60–70% of these advanced cases, but still less than 5% of all TCs [3], RAI treatment becomes ineffective and the overall survival rate at 10 years drops to less than 10% [4]. Other histological types are much less common but have a more aggressive nature, like Hürthle cell carcinoma and poorly differentiated carcinoma (PDTC), which account for 2% and 5% of TC, respectively [3]. Compared to DTC, PDTC is characterized by a higher risk of persistence/recurrence (both at local and distant sites), and by a higher mortality rate with a median survival rate of 6 years [5,6]. Anaplastic thyroid carcinoma (ATC), the most aggressive among thyroid tumors, represents only 1% of all TCs, but accounts for the majority of all TC deaths and has a median survival of 6 months [6]. This tumor is unable to take up RAI and has had no chance of cure so far [6,7]. Medullary thyroid carcinoma (MTC), which arises from neural crest C cells, accounts for about 3–5% of all TCs and has a much worse prognosis than DTC, with a 10-year survival rate of 50%, which becomes even lower in patients with advanced disease at the time of diagnosis [8].

Until recently, there were few effective treatments such as radiotherapy, chemotherapy or local therapies in patients with advanced TC and unresectable local or metastatic disease. However, in the last decade the paradigm of TC therapy has been revolutionized by the introduction of systemic therapies with multikinase inhibitors (MKIs) and, more recently, with next-generation targeted therapies [9]. Among the MKIs approved by the Food and Drug Administration (FDA) and by the European Medical Agency (EMA), lenvatinib and sorafenib are currently used in daily practice for the treatment of advanced RAI-refractory DTC, while vandetanib and cabozantinib are used for the treatment of advanced MTC. Cabozantinib has also been recently approved as second-line therapy for advanced RAI-refractory DTC. These MKIs inhibit specific oncogene alterations and have a good efficacy on progression-free survival (PFS) [10,11,12,13,14]. These molecules also inhibit other “off-target” tyrosine-kinase receptors (e.g., the vascular endothelial growth factor receptor—VEGF-R) causing several adverse events (AEs) and significantly impairing the patients’ quality of life (QoL) [7]. The strong impact on QoL often requires dose reduction or periodic discontinuations of the drug [15]. More recently, a new generation of agents acting against specific mutated oncogenes has been developed. These drugs include selpercatinib and pralsetinib, highly selective for gene fusions and point mutations of the protooncogene rearranged during transfection (RET) [16,17], and they have shown a good efficacy on Objective Response Rate (ORR) and on PFS in phase-2 trials. They also have a limited toxicity profile due to their high molecular specificity, so that the results of the ongoing phase-3 trials are very much awaited [6]. Other molecules with different targets are under investigation, for example the combination of dabrafenib and trametinib, which has been approved for the treatment of ATC with BRAFV600E somatic mutation that produces a dual inhibition of BRAF and MEK [18]. Larotrectinib and entrectinib, both able to inhibit the NTRK fusion gene, are also under investigation for their employment in the treatment of TC harboring this somatic alteration [19,20]. 

The management of patients treated with these new therapies has forced physicians to deal with new side-effects, in some cases very different from the classic ones of antiblastic therapy. The toxicity profile is generally milder than with traditional chemotherapy, but still present. Some of the most common adverse events (AEs) during therapy with MKIs can severely affect the nutritional profile, and include weight loss, nausea, anorexia, stomatitis, diarrhea, which represent a major issue in the management of these patients [15]. These AEs may be associated with malnutrition and cancer-related cachexia, which characterize the advanced disease stage and may be present before starting these treatments or may develop afterwards. To our knowledge, no prospective studies have demonstrated an effect of nutritional therapy on the outcome of patients with advanced TC which has instead been suggested in other type of tumors [21]. Recently, an interesting study showing a prognostic predictive role of the CONUT score, an immune-nutritional screening score calculated taking into account albumin, cholesterol and lymphocytes, in patients with advanced TC has been published [22]. Moreover, it was observed that sarcopenia is a predictive factor for MKIs treatment outcome in metastatic TC [23]. 

The aim of this review was to discuss the role of nutritional therapy in advanced TC and the importance of an early recognition and careful management of malnutrition and cachexia during systemic therapy with MKIs.

## 2. Malnutrition, Cancer-Related Cachexia and Thyroid Carcinoma

Malnutrition is common in cancer patients and is caused by a compromised intake or assimilation of nutrients, which may be connected either to the cancer itself or to its specific treatments [24]. Malnutrition can also lead to an impairment of the QoL and can worsen the toxicity of treatments. It is observed that up to 10–20% of deaths in cancer patients are consequences of malnutrition rather than the cancer itself [25]. Malnutrition can result in cachexia, one of the most severe manifestations of cancer. The European Society for Clinical Nutrition and Metabolism (ESPEN) defines cancer cachexia as a multifactorial syndrome leading to progressive functional impairment characterized by loss of skeletal muscle mass, with or without loss of fat mass, which is not fully reversible with conventional nutritional support [26]. The prevalence of cancer-related cachexia depends on the type of cancer in question. Head and neck cancers are commonly associated with this condition [27], most likely because of the direct effects on food intake, digestion, and absorption of nutrients, but also because of other risk factors such as advanced cancer stage, specific tumor characteristics of aggressiveness, male sex, advanced age, genetic risk factors, and comorbidities [27]. Importantly, drugs like MKIs, which are used to treat advanced TC, have proved to exert catabolic effects on the skeletal muscle [27,28]. Unlike simple malnutrition, negative protein intake and negative energy balance in cancer patients have a multifactorial etiology resulting from unintentional decreased food intake, systemic inflammation, and abnormal metabolism [29]. Primary anorexia (i.e., reduced or total loss of appetite) is often present and is controlled by the central nervous system probably due to an inflammation-driven resistance of the hypothalamus responding to orexigenic and anorexigenic signals [30]. Anorexia can also be secondary to sign/symptoms of the neoplastic disease or to the AEs of treatments (i.e., MKIs, External Radiotherapy), such as chemosensory disturbances in taste and smell, stomatitis, xerostomia, poor dentition, reduced upper gastrointestinal motility, distal tract dysmotility and uncontrolled pain [26]. All together these symptoms can determine nausea, vomiting, early satiety, diarrhea or constipation thus severely contributing to loss of weight.

In these patients, energy intake is typically lower than energy requirements, which are increased because of systemic inflammation and related metabolism impairment. A plethora of molecules such as pro-inflammatory cytokines, eicosanoids, heat shock proteins, members of the transforming growth factor-β (TGFβ) superfamily, are secreted from the tumor itself because of tumor-host interaction. These molecules can stimulate catabolism in several target organs, primarily the skeletal muscle and the adipose tissue. Murine models display an increased rate of whole-body glycolysis and gluconeogenesis from the Cori cycle, as well as higher triacylglycerol or fatty acid cycling, with an excessive mobilization of lipids [27]. Inflammation mediators also act in the central nervous system by stimulating nausea, anorexia, fatigue, and release of adrenal corticosteroids via the hypothalamus-pituitary-adrenal axes [29].

Moreover, a condition of negative energy balance (i.e., energy expenditure exceeds caloric intake)” is partially connected with the tumor metabolism, which competes with other organs and tissues for energy substrates. Tumor tissue also has an inner metabolic rate, proportional to the mass and degree of aerobic metabolism on which the cancer cells are mostly dependent, according to the “Warburg effect” [28,31]. This inefficient way of generating energy produces heat instead of ATP synthesis, a process that further contributes to cachexia [32].

These processes all result in muscle protein depletion and sarcopenia, which are key features of cancer-associated cachexia. It has been demonstrated that weight loss, low muscle index, and low muscle attenuation are independent risk factors of survival, regardless of overall body weight [33]. Skeletal muscle depletion is also associated with physical impairment [34], loss of strength, increased risk of falls, impaired respiratory function, post-operative complications, chemotherapy toxicity [35,36]. 

## 3. Adverse Events Affecting Nutritional Profile during Therapy with MKIs

In patients with advanced TC, cachexia and sarcopenia may be caused by both neoplastic disease and MKI systemic therapy. As multitarget inhibitors, these molecules act against different tyrosine-kinases, such as VEGFR1, VEGFR2, VEGFR3, fibroblast growth factor receptors (FGFRs 1–4), platelet-derived growth factor receptors (PDGFR), RET and c-KIT proto-oncogenes [37]. The inhibition of these off-targets is responsible for AEs, most of which are related to the nutritional profile [15]. Table 1 shows the most common AEs of the MKIs approved for clinical practice in the treatment of advanced RAI-refractory DTC and advanced MTC. In particular, symptoms involving the gastrointestinal tract are more frequent, especially if pulled together, compared to others such as hypertension, hand-foot syndrome or QT prolongation. MKIs are known to have a direct effect on inhibiting muscle protein synthesis [38], but they can also cause asthenia, which leads to increased immobilization. Indeed, fatigue contributes to the reduction of physical activity, worsening the condition of the skeletal muscle [39]. For this reason, the European Thyroid Association (ETA) recommends the adoption of some strategies, such as regular physical activity; MKI intake in the evening; monitoring of electrolytes, hemoglobin, TSH, cortisol; hydration maintenance; limited caffeine intake; adequate food assumption [3]. These strategies, all included in the new concept of pre-habilitation practice, should be started from the beginning of the therapy or even earlier to prevent fatigue and anorexia [3]. The progression toward malnutrition and sarcopenia should be slowed down as much as possible, as it is known that advanced TC patients with sarcopenia treated with MKIs present a worse prognosis than those without sarcopenia in terms PFS [23,40,41]. To obtain the greatest benefit from systemic therapy, the control and prevention of AEs, even changing diet, is essential since it allows treatment continuation at a lower daily dose, but without interruptions, which are correlated with lower efficacy [15,42]. 

## 4. Nutritional Intervention

### 4.1. Goals of Nutritional Therapy

Nutrition plays a crucial role in the multidisciplinary treatment of advanced TC and should be taken into consideration from the moment of its diagnosis, integrated by the assessment of the nutritional status of the patients during antineoplastic treatments [43].

Nutritional intervention comprises nutritional screening, assessment, and therapy. This therapeutic approach aims at identifying, preventing and treating cancer-related malnutrition, if necessary with oral nutritional supplements or through either enteral (EN) or parenteral (PN) artificial nutrition [43]. An adequate intervention might help to delay weight loss and progression toward sarcopenia, and it might improve nutritional parameters and body composition. Furthermore, it can mitigate or counteract some symptoms with nutritional impact that are related to the disease or to the side-effects of the systemic MKI therapies, thereby improving QoLand survival. Early and intensive medical nutrition therapy has been demonstrated to be beneficial in improving several treatment outcomes in patients affected by by different cancers (e.g., head and neck, lung, breast, ovary, colorectal, upper gastrointestinal, leukemic) and undergoing chemotherapy and radioterapy treatment [36]. Similarly, more recent studies have proposed to tackle malnutrition and cachexia to improve cancer prognosis in MKI patients. In a study of 297 patients treated with sorafenib for hepatocarcinoma, pre-sarcopenia was a significant prognostic factor of worse overall survival [41]. Similarly, in metastatic renal cell carcinoma treated with sunitinib, sarcopenia was found to be independently associated with shorter PFS [44] also in patients with advanced TC under lenvatinib or vandetanib therapy [23].

### 4.2. Nutritional Screening

Nutritional screening aims at stratifying the risk of malnutrition and should be performed as early as possible, certainly prior to MKI treatment. The nutritional status should also be reassessed during follow-up [45]. For this purpose, the ESPEN guidelines recommend the evaluation of body mass index (BMI) and of weight loss in combination with the measurement of caloric intake, by using validated tools [43] such as NRS-2002 (Nutrition Risk Screening 2002), MUST (Malnutrition Universal Screening Tool), MST (Malnutrition Screening Tool), or the Mini Nutritional Assessment Short Form Revised [46]. When considered alone, BMI has low sensitivity in detecting changes in the nutritional status, especially in obese patients. Low BMI and concomitant weight loss history have been considered useful indicators to identify the presence of nutritional risk. However, the reduction of hunger sensation may occur irrespective of weight loss and anorexia, and it should be included in the screening test, as it is an early indicator for the development of malnutrition [47].

### 4.3. Nutritional Assessment

As recommended by ESPEN, in case of increased risk of malnutrition, a complete assessment of the nutritional status should be performed: Subjective Global Assessment (SGA) and Patient Generated-Subjective Global Assessment (PG-SGA) allow to collect data for a detailed nutritional assessment of cancer patients in both outpatient and inpatient settings [46,48,49,50]. This procedure includes the assessment of anthropometric measurements (weight, height, and BMI), weight loss (>5% in the last six months) and body composition, loss of muscle mass and/or subcutaneous fat, presence of pressure ulcers, presence of nutrition impact symptoms, inflammatory markers and food habits. Accurate evaluation of a patient’s habits is necessary, and it should investigate the usual energy and macronutrient intake and verify their adequacy. As regards the quantification of changes in dietary intake, a reduction of 50% of weight (maintaining energy for more than 1–2 weeks and/or a possible malabsorption requiring a longer period of time) are factors that might increase the risk of malnutrition [43]. 

In response to the lack of consensus on the most appropriate assessment of the nutritional status, the Global Leadership Initiative on Malnutrition (GLIM) has recently developed a model for the diagnosis of malnutrition, adding new criteria to the ones used by ESPEN [24]. According to the GLIM model, the diagnosis of malnutrition requires at least one phenotypic criterion (e.g., involuntary weight loss, low BMI, reduced muscle mass) and one etiologic criterion (e.g., reduced food intake or absorption, inflammation or comorbidity) [24,45]. The phenotypic criteria may also allow the staging of malnutrition [24], as shown in Table 2. 

Computed Tomography, Magnetic Resonance Imaging and dual energy X-ray absorption are considered the gold standard techniques for the assessment of body composition and skeletal muscle depletion. However, these methods are expensive and require technicians with a high level of expertise. In daily clinical practice it is suggested to assess anthropometric measures that can be applied easily and at low cost [51]. Physical performance may be graded by using the ECOG scale [49], or the Karnofsky Performance Scale [52]. The presence of systemic inflammation may be assessed by measuring serum albumin, complete blood count, lymphocyte count, C reactive protein, transferrin and fibrinogen [45,53]. 

### 4.4. Nutritional Requirements

The regulation of body weight is controlled by the equilibrium of the energy balance equation, which comprises energy intake and daily energy expenditure. In patients with cancers, before starting a nutritional intervention it would be useful to measure the 24 h energy expenditure. This comprises different components, such as resting metabolic rate (RMR), thermic effect of food (TEF), and energy cost of physical activity. In patients with advanced cancers, it has been hypothesized that the Resting Energy Expenditure (REE) might be high despite the concomitant reduced physical activity [53,54,55,56]. When a nutritional intervention is required, the dietitians should consider those patients as healthy subjects in terms of energy metabolism. The caloric intake should be set between 25 and 30 kcal/kg of body weight, taking into account patients’ age and gender, balancing the non-protein caloric intake between carbohydrates and fats or, in the presence of insulin resistance, increasing the ratio of fats and carbohydrates to reduce the glycemic load [25].

In a study of 297 cancer patients, the average measured REE (1533 kcal/day) was higher than the predicted one (1380 kcal/day) [57]. Interestingly, no difference in absolute energy intake was observed between weight-losing and weight-stable patients. However, this latter group displayed higher caloric intake per unit of body weight, suggesting that weight loss might not depend on a reduction in caloric intake, but rather on increased REE. This result might indicate that the compensatory mechanism leading to an increased food intake as a response to the rise in REE can be lost in patients with cancers.

As concerns protein intake, the ESPEN practice guidelines recommend a protein intake greater than 1 g/kg/day, which can increase up to 1.5 g/kg/day. However, a higher protein intake may be required in case of severe free mass loss [25,47,58].

Therefore, one of the most important goals in the nutritional approach is to teach patients to increase the energy intake with food items rich in protein. Supplementation with amino acids including branched-chain amino acids (i.e., leucine, isoleucine and valine, β-hydroxy β-methyl butyrate, carnitine and creatine) was also tested in cancer patients [43] in an attempt to optimize the nutritional status by counteracting muscle atrophy. However, further studies are necessary to demonstrate the beneficial effects of this approach.

Studies on vitamin supplementation have shown that advanced cancer patients with adequate food intake do not show any differences compared to healthy control patients [25], suggesting that vitamin requirement can be achieved with a daily diet. However, in case of inadequate food intake due to appetite reduction, oral, EN or PN supplements might ensure the same vitamin intake as occurs in the general population.

**Figure 1:**
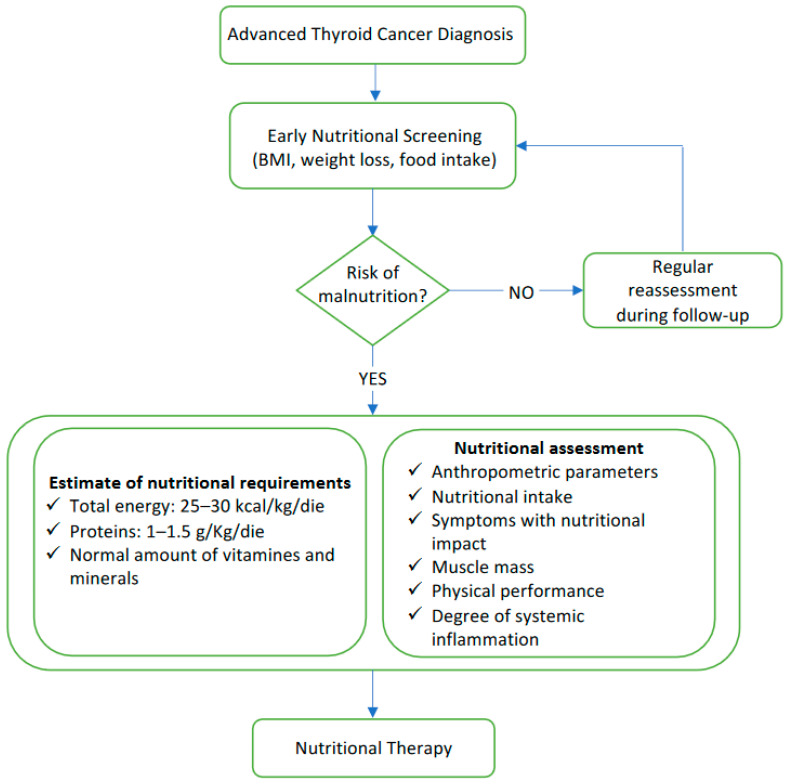
Flow-chart summarizing the main stages of nutritional screening and nutritional assessment.

The main stages of the nutritional screening and assessment in patients with advanced cancer to be performed both before and during the treatment with TKIs are reported in Figure 1.

### 4.5. Nutritional Counseling

Nutritional counseling is considered the preferred approach to maintain or improve nutritional status, which is altered on account of the altered nutritional demand. The aim of nutritional counseling is to address the altered nutritional demands through [36] a diet enriched in energy and protein, including regular food or fortified foods such as meals or snacks, monitoring compliance with nutritional intervention and adapting the strategy if necessary [47].

Several guidelines report nutritional counseling as a standard of care for patients who are malnourished or at risk of malnutrition [36,46,47,59,60,61].

Oral nutrition represents a significantly important moment of the daily routine, to be spent with family and friends, avoiding the tendency to isolation, and this contributes substantially to the autonomy of the patient [25]. The perception that the prescribed diet is specific, customized, and suitable to individual needs, gives the patient a feeling of control. The diet is indeed the only aspect that the patient feels he can control throughout the course of treatments and interventions. Adequate food intake, recognized by patient, family members and caregivers, is crucial to maintain daily activity and functional capacity, and to make treatments more effective. All these factors can potentially help to ameliorate the QoL of the patient [43].

## 5. Nutritional Therapy

**Figure 2:**
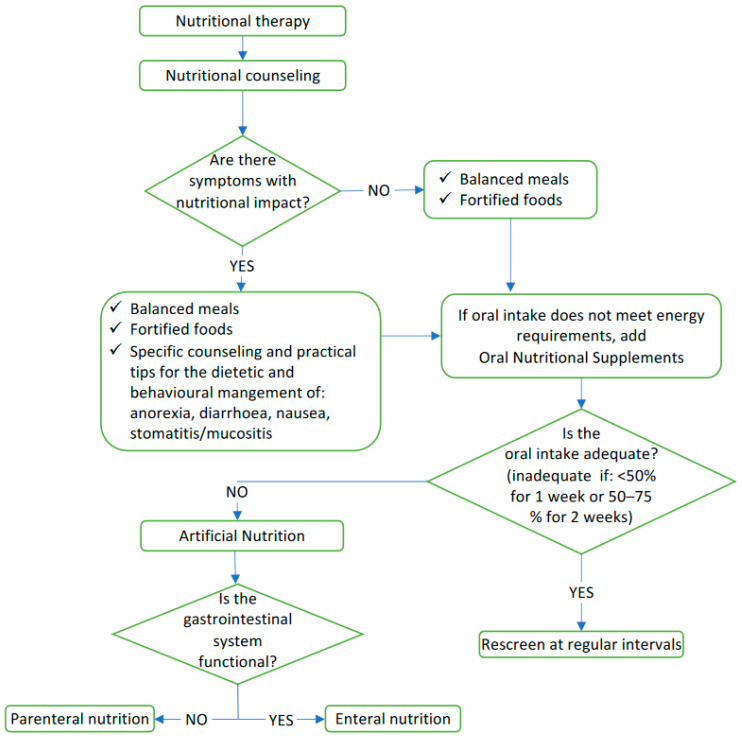
Decisional algorithm for nutritional therapy.

Nutritional therapy needs to be started when patients are not yet severely malnourished and it comprises dietician-aided dietary counseling that is aimed at improving spontaneous food intake, oral supplementation with oral nutritional supplements (ONS) or, in more severe cases, EN or PN [43,49], as described in detail in Figure 2.

### 5.1. Dietetic and Behavioral Management of AEs with Nutritional Impact

Adult patients with advanced TC treated with MKIs may experience nutritional impact side-effects including diarrhea, anorexia, dysgeusia, mucositis/stomatitis, nausea, which require nutritional interventions that include specific behavioral dietary measures.

Diarrhea is frequent and, together with anorexia, it contributes to fat and muscle loss [39]. In its initial stages it can be managed with dietary adjustments, for instance by drinking adequate amounts of liquid at room temperature, by excluding caffeinated drinks, and by limiting sparkling drinks. Patients can also benefit from practical measures such as small and frequent meals, avoiding fried, fatty foods and reducing fiber-rich ones such as whole grains, bran, vegetables, and peeled fruits. They should limit dairy products in case of lactose intolerance [53]. Fluid and electrolyte replacement, associated with pharmacological interventions (e.g., loperamide, amisulpride, codeine) may be necessary in cases of more severe and persistent diarrhea [3].

Mucositis and stomatitis can also occur, usually within the first 2–4 weeks of therapy, and can exacerbate anorexia. A diet based on soft and slippery foods cut into small pieces, chopped or smoothed if necessary, can facilitate nutrition in presence of mucositis/stomatitis, while spicy, salty or acidic foods should be limited [53]. It is recommended to keep a good oral hygiene with frequent mouthwash using bicarbonate or high-molecular-weight hyaluronic acid, or aloe vera extracts, and to recur to topical analgesics (lidocaine 2%, diphenhydramine, bismuth subsalicylate, aluminum or magnesium hydroxide) in more severe cases [3].

In case of anorexia, it is useful to direct the patient towards the choice of high-concentration caloric-protein foods, to plan meals and to always eat one’s favorite foods, either cooked or frozen, and to ask for support in the preparation of meals since the smells of the kitchen can sometimes interfere negatively with the desire to eat.

In the presence of nausea, in addition to the advice that can be given for anorexia, it may be useful to direct the patient towards crisp and salty foods, while avoiding fat and spicy ones. In some cases cold dishes may be better than warm ones [53].

Advanced TC patients may benefit from adjuvant treatment with external beam radiotherapy (EBRT). This technique can be used to treat inoperable primary or locally recurrent TC, to treat recurrent lymphnode metastases or bone metastases. EBRT allows to control the disease and to improve outcome [62,63]; on the other hand, it may cause local AEs mainly on the mucosal tract. Such events include dysphagia, dysgeusia and mucositis, which may worsen the loss of weight caused by other concurrent therapies [45]. If dysphagia occurs, it is necessary to change the consistency of foods according to the patient’s swallowing function to limit the risk of ab ingestis pneumonia. In case of dysphagia the diet for the patient must be safe but at the same time pleasant, and able to provide a sufficient protein caloric intake.

### 5.2. Oral Nutrition Supplement

The additional use of Oral Nutrition Supplement (ONS) must be considered when an enriched diet is not efficient to reach the nutritional goals [25]. ONS are characterized by low volumes, a known nutritional composition, and a high protein caloric concentration. They contain vitamins and trace elements in balanced quantities and have variable consistency adapting to the specific needs of the patient. Supplementation with ONS can generally provide a daily intake of up to 600 kcal. However, supplementation with ONS should be accompanied by specific indications regarding their intake to promote tolerance and regular use.

Several ONS products are available but the choice depends on patient preference, consistence deeding, current macro- and micro-nutrient intake and local policy [61].

Several studies evaluating nutritional counseling with and without the use of ONS have shown improvements in nutritional outcomes with weight gain, increased BMI and better scores on a validated nutritional assessment test (e.g., patient-generated subjective global assessment, PG-SGA) [64].

### 5.3. Artificial Nutrition

When oral nutrition remains inadequate despite nutritional interventions (e.g., counseling and ONS) implementation with EN is recommended. From a practical point of view, if energy intake is less than 50% of the requirement for more than one week, or only 50–75% of the requirement for more than two weeks, EN should be started. Even in the case of severe side-effects such as fistulization, either tracheal or oesophageal fistulae, or intestinal perforation, hospitalization is needed to provide artificial nutrition for the patient [65].

If EN is insufficient or impracticable, PN is required, in order to stabilize the patient’s nutritional status [66].

One of the most serious risks during artificial nutrition can be the “refeeding syndrome”, which is defined as a variation in the balance of body fluids and electrolytes due to hormonal and metabolic changes related to nutrient intake. These changes can occur in malnourished patients when oral, EN or PN nutrition is begun too aggressively after a period of inadequate nutrition [67]. Such conditions can be avoided by gradually increasing protein caloric intake, and by monitoring the electrolytes and the phosphate values in the blood [68].

As a general concept, the possible physiological and/or psychological benefits need to be evaluated in patients with short-term life expectancy (i.e., less than 2 months) for whom the risks of a PN might be higher than the advantages.

### 5.4. Others Dietary Approaches

There are no known diets that are effective in treating cancer or preventing its recurrence. Dietary approaches not based on clinical trials, whose efficacy has not been demonstrated, could be potentially harmful, and therefore contraindicated [25].

The implementation of diets limiting intake energy is not recommended in malnourished patients nor in those at risk of malnutrition since they increase the risk of insufficient energy, of fat and protein intake, and of micronutrient deficiency. As concerns the ketogenic diet, no clinical studies have demonstrated that it produces benefits in patients with cancer. Owing to their low palatability, ketogenic diets might lead to an insufficient energy intake and to further weight loss [25].

No specific suggested or prohibited foods are related to the thyroid involvement, either in patients treated with thyroidectomy which represents the majority of cases, or in the few who could not undergo this surgical procedure.

## 6. Conclusions

In the multidisciplinary management of patients with advanced TC, it is necessary to include an early and periodic evaluation of the nutritional status since these patients are at risk of severe malnutrition and cachexia. The systemic therapy with MKIs, although effective in controlling the neoplastic disease, may expose the patient to AEs with nutritional impact that may represent a further risk of malnutrition and worse prognosis. Weight loss, anorexia, nausea, diarrhea, stomatitis are common causes of drug dose reduction or discontinuation. It is therefore important to prevent these side-effects and to provide an early treatment to improve the QoL and the prognosis of patients.

Nutritional screening should be performed before starting systemic therapy and regularly during follow-up. Whenever a condition of risk is identified, the patient should be submitted to specific assessment with the estimate of energy requirements followed by nutritional counseling and, if necessary, by nutritional therapy. As a matter of fact, the diet is the only factor that patients feel they can actively control throughout the course of treatment. A good nutritional status allows the maintenance of daily functional activity and a better tolerance of the treatments.

## Figures and Tables

**Table 1:** Most common side-effects of multikinase inhibitors used in advanced thyroid cancer.

Adverse Events (%)	Lenvatinib [11]	Sorafenib [14]	Vandetanib [12]	Cabozantinib [13]
Hypertension	68	41	32	33
Diarrhea	59	69	56	63
Anorexia	49	32	21	46
Fatigue	59	50	24	41
Nausea	41	20	33	43
Weight loss	46	47	10	48
Mucositis/stomatitis	36	23	NR	62
Hand-foot Syndrome	32	76	NR	50
Hypertension	68	41	32	33
Diarrhea	59	69	56	63
Anorexia	49	32	21	46
Fatigue	59	50	24	41
Nausea	41	20	33	43
Weight loss	46	47	10	48
QT prolongation	8	NR	14	NR

NR: not reported.

**Table 2:** Staging of malnutrition based on phenotypic criteria.

	Stage 1 Moderate	Stage 2 Severe
Weight loss (%)	5–10% within the past 6 months or 10–20% beyond 6 months	>10% within the past 6 months or >20% beyond 6 months
BMI (kg/m²)	<20 kg/m^2^ if <70 years, or <22 kg/m^2^ if >70 years	<18.5 kg/m^2^ if <70 years or <20 kg/m^2^ if >70 years
Muscle Mass	Reduced muscle mass determining mild-to-moderate functional deficit	Reduced muscle mass determining severe functional deficit

BMI: Body Mass Index.

## Data Availability

Not applicable.
